# Eccentric treadmill training and skeletal muscle immunometabolic responses in HFD-induced insulin resistance

**DOI:** 10.3389/fimmu.2026.1757925

**Published:** 2026-02-25

**Authors:** Wei Luo, Yuanyuan Wang, Siyi He, Shijin Zhao, Yue Zhou, Lei Ai

**Affiliations:** 1 School of Sport and Health, Nanjing Sport Institute, Nanjing, China; 2 School of Kinesiology, Beijing Sport University, Beijing, China; 3 Research Center for Kinesiology, Jiangsu Research Institute of Sports Science, Nanjing, China

**Keywords:** AKT signal, eccentric exercise, insulin resistance, macrophages, skeletal muscle

## Abstract

**Introduction:**

In recent years, repeated eccentric exercise has gained increasing attention as a potentially superior intervention for ameliorating insulin resistance (IR). However, the underlying mechanisms responsible for these effects remain incompletely understood. This study aims to investigate the effects and underlying mechanisms of moderate-intensity eccentric treadmill training on skeletal muscle IR.

**Methods:**

A mouse model of IR was established using a high-fat diet (HFD) for 12 weeks, followed by an 8-week eccentric treadmill training. *In vitro*, RAW264.7 macrophages under high-glucose conditions were treated with the AKT agonist SC79 or inhibitor MK2206. Comprehensive assessments included protein localization and expression (immunofluorescence, Western blot), macrophage polarization status (flow cytometry), inflammatory infiltration (H&E staining), cytokine profiles (ELISA), and cellular viability (CCK-8).

**Results:**

HFD-induced IR led to elevated pro-inflammatory factors and reduced GLUT4, F4/80 & phospho-AKT (Ser^473^) co-localization, IL-10, and Arg-1 levels, all of which were significantly reversed by eccentric training. *In vitro*, high glucose reduced the phospho-AKT (Ser^473^)/AKT ratio, while SC79 suppressed an M1-like pro- inflammatory phenotype, as indicated by decreased iNOS and F4/80&CD86 double-positive rates and increased Arg-1 and F4/80&CD206 double-positive rates. These effects were abolished by MK2206.

**Conclusion:**

Moderate-intensity eccentric treadmill training ameliorates HFD-induced skeletal muscle IR, likely driven by AKT-mediated reduction in pro-inflammatory M1 macrophage polarization.

## 1 Introduction

Insulin resistance (IR) constitutes a common pathogenic foundation for a range of chronic metabolic diseases (1, 2). It is characterized by diminished insulin-activated glucose transport and metabolism in target cells, ultimately resulting in hyperglycemia (3, 4). As the largest metabolic organ in the human body, skeletal muscle plays a vital role in the maintenance of metabolic homeostasis. Skeletal muscle accounts for approximately 80% of insulin-mediated glucose uptake, thereby representing the primary site for the development of IR (5, 6). Therefore, investigating lifestyle interventions—particularly exercise—and their underlying mechanisms for improving skeletal muscle IR is of considerable importance for the early prevention and management of metabolic disorders.

In recent years, eccentric exercise has gained increasing attention in clinical rehabilitation as an effective intervention for delaying and ameliorating metabolic diseases (7–10). Numerous studies have demonstrated that, under equivalent cardiovascular load, eccentric muscle contractions enable skeletal muscle to perform a greater volume of work, thereby imposing a more substantial physiological stimulus (11, 12). This suggests that eccentric exercise may represent a more effective modality for improving skeletal muscle IR. Although unaccustomed acute eccentric exercise can induce ultrastructural damage to skeletal muscle and lead to delayed onset muscle soreness (DOMS), repeated bouts of eccentric exercise at the same intensity significantly alleviate DOMS and accelerate recovery-a phenomenon termed the Repeated Bout Effect (RBE) (13, 14). These findings indicate that repeated moderate-intensity eccentric exercise may constitute a safer and more effective exercise strategy for populations with metabolic diseases. Nevertheless, the underlying mechanisms through which moderate-intensity eccentric exercise improves IR remain incompletely understood.

Macrophages, as crucial immune cells, exhibit remarkable plasticity (15). Their ability to polarize into functionally distinct phenotypes—pro-inflammatory M1 or anti-inflammatory M2—in response to different microenvironments, thereby regulating the inflammation-metabolism axis, has recently established them as a research focus in metabolic diseases (16, 17). Our previous studies demonstrated enhanced M1 macrophage polarization in insulin-resistant adipose tissue (18), and similarly, high-glucose treatment *in vitro* induced M1 polarization in macrophages (19). Moreover, substantial evidence confirms that uncontrolled M1 macrophage polarization in skeletal muscle triggers chronic inflammation and disrupts metabolic homeostasis, whereas M2 polarization exerts anti-inflammatory effects and promotes metabolic balance restoration (20, 21). Notably, compared to the inflammatory response elicited by a single bout of acute eccentric exercise, repeated eccentric exercise results in increased macrophage infiltration in skeletal muscle alongside an attenuated inflammatory response (22–24). The observed adaptive shift implicates macrophages as potential key mediators through which moderate-intensity eccentric exercise accelerates skeletal muscle repair and ameliorates IR, a premise that awaits definitive validation.

The Protein Kinase B (AKT) signaling pathway represents a key regulator of macrophage polarization and plays a crucial role in macrophage activation and phenotypic determination (25–27). However, the involvement of AKT signaling in mediating the effects of moderate-intensity eccentric exercise on macrophage polarization remains incompletely elucidated. To address this gap, the present study employed a moderate-intensity downhill treadmill running protocol (at a -5° incline) in a mouse model of IR. By focusing on skeletal muscle macrophages and their AKT signaling activity, this research aimed to systematically investigate the therapeutic effects of downhill treadmill training on skeletal muscle insulin sensitivity in IR mice and to explore the underlying molecular mechanisms. This work is expected to advance current understanding of eccentric exercise in the prevention and management of metabolic diseases, provide biological validation for its therapeutic application, and establish a theoretical foundation for developing exercise prescriptions targeting IR and type 2 diabetes.

## 2 Materials and methods

All experimental procedures in this study were performed in strict accordance with the Laboratory Animal-Guideline for Ethical Review of Animal Welfare of China and received approval from the Laboratory Animal Ethics Committee of Nanjing Sport Institute (Approval code: GZRDW-2022-02).

### 2.1 Animals and feeding

Fifty-two 5-week-old male C57BL/6N mice were procured from Charles River Laboratories (Beijing, China). Following a one-week acclimatization period, the mice were randomly allocated into two groups: a normal standard diet group (NSD, n = 26) and a high-fat diet group (HFD, n = 26). The NSD group received a standard diet (1022, Huafukang, Beijing, China), whereas the HFD group was fed a high-fat diet (H10060, Huafukang, Beijing, China). The dietary intervention was maintained for 12 weeks, with weekly monitoring of fasting blood glucose (FBG) concentrations. Subsequently, 12 mice were randomly selected from each group (NSD and HFD) for further metabolic evaluations, including fasting insulin (FINS) measurement, intraperitoneal glucose tolerance test (GTT), and insulin tolerance test (ITT). IR was assessed using the homeostasis model assessment of IR (HOMA-IR), calculated as [FBG (mmol/L) × FINS (μU/mL)]/22.5, and insulin sensitivity was evaluated via the insulin sensitivity index (ISI), determined as 1/(FBG × FINS). The number of animals assigned to each group is calculated with power analyses using means of fasting insulin levers in our preliminary study: 0.05 as significance criterion (two-tailed), 4.3 as expected difference, 3.7 as estimated SD and 0.8 as desired power, resulting in a minimum required sample size of n = 10 per group.

For the GTT, after a 12-hour fast (with free access to water), the mice were administered an intraperitoneal injection of 20% glucose solution (2 g/kg body weight). Blood glucose levels were measured at 30-, 60-, 90-, and 120-minutes post-injection, and the area under the curve (AUC) was calculated. For the ITT, following a 4-hour fast (water provided ad libitum), the mice received an intraperitoneal injection of insulin (0.75 U/kg body weight). Blood glucose levels were assessed at 15, 30, 60, and 120 minutes after injection, and the corresponding AUC was computed.

### 2.2 Exercise protocol and grouping

Following successful model establishment, 6 mice were randomly selected from both the NSD and HFD groups to undergo a maximal exercise capacity test. Prior to testing, all animals completed a three-day acclimatization period on the motorized treadmill. The test began at an initial speed of 10 m/min and a slope of -5°, maintained for 5 minutes. The speed was then increased to 13 m/min, with subsequent increments of 3 m/min every 3 minutes until exhaustion, while the slope remained constant at -5°. The maximum exercise intensity achieved was used to determine the intensity for the subsequent exercise intervention (19). The measured maximal running speeds were 47.5 ± 1.6 m/min for the NSD group and 43.5 ± 2.3 m/min for the HFD group.

For the formal exercise intervention, the mice were randomly divided into four groups: NSD sedentary (NC, n = 13), NSD exercise (NE, n = 13), HFD sedentary (OC, n = 13), and HFD exercise (OE, n = 13). The sedentary groups were maintained under their original housing conditions without additional exercise. The exercise groups performed moderate-intensity eccentric treadmill running at 45% of their respective maximal running speeds-specifically, 21.4 m/min for the NE group and 19.6 m/min for the OE group—at a constant slope of -5°. The training was conducted for 1 hour per day, 6 days per week, over a period of 8 weeks (19, 28, 29).

### 2.3 Sample collection

**Figure 1:**
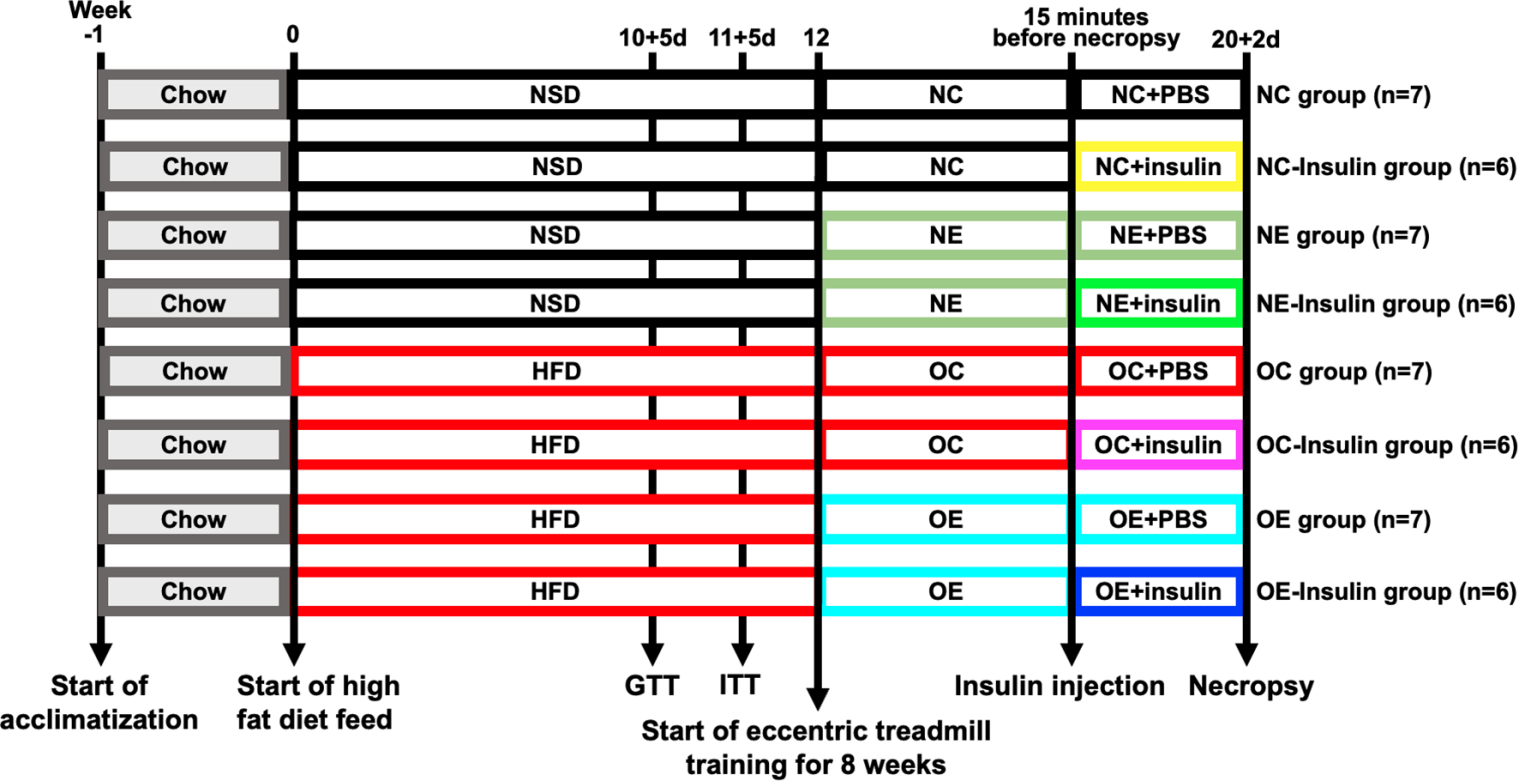
Overall design of this study. After 1 week of acclimatization, mice were fed a normal standard diet (NSD group) or high-fat diet (HFD group) for 12 weeks. After 12 weeks of diet, 12 mice in each group were randomly selected for further metabolic evaluations. Following successful model establishment, the mice were randomly submitted to the eccentric treadmill training protocol for 8 weeks, which were randomly assigned to four groups: NSD sedentary (NC), NSD exercise (NE), HFD sedentary (OC), and HFD exercise (OE). Six randomly selected mice received an intraperitoneal injection of insulin 15 minutes before sampling from each group. Serum samples were obtained at 12 and 20 weeks, muscle samples were obtained at 20 weeks.

At 20 weeks, after an 8-week period of eccentric treadmill running, animals in the fasting state (12 h) were deeply anesthetized 48 h after the last exercise session, samples were collected. From each group, six randomly selected mice received an intraperitoneal injection of insulin (0.75 U/kg body weight) 15 minutes before sampling (NC-Insulin, n = 6; NE-Insulin, n = 6; OC-Insulin, n = 6; OE-Insulin, n = 6), while the remaining mice were injected with an equal volume of PBS as a control (NC, n = 7; NE, n = 7; OC, n = 7; OE, n = 7). The number of animals assigned to each group is calculated with power analyses using means of skeletal muscle IL-10 contents in our preliminary study: 0.05 as significance criterion (two-tailed), 6.7 as minimum expected difference, 5.8 as estimated SD and 0.8 as desired power, resulting in a minimum required sample size of n = 6 per group. During sampling, blood was collected via eyeball enucleation, and bilateral gastrocnemius muscles from the hind limbs were dissected. Connective tissues such as tendons were carefully removed with a surgical scalpel. One side of the muscle was rinsed with PBS, rapidly frozen in liquid nitrogen, and stored for subsequent protein extraction and analysis. The contralateral muscle was embedded in embedding medium for the preparation of frozen sections, which were used for subsequent pathological and immunofluorescence staining. Time points of experimental procedures are illustrated in **Figure 1**.

### 2.4 Cell culture and grouping

The murine macrophage cell line RAW264.7 was obtained from the Cell Bank of the Chinese Academy of Sciences and randomly divided into two groups: control group (Con group) and 60 mmol/L D-glucose group (60mM group). The 60 mmol/L L-glucose was used in Con group for the high glucose as an osmotic control for 60 mmol/L D-glucose. SC79 (HY-18749, MCE, USA) and MK2206 (HY-10358, MCE, USA) were used as a specific AKT agonist and inhibitor, respectively. The working concentrations applied in the experiments were 10 μg/mL for SC79 and 1 μmol/L for MK2206. Macrophages were treated for 3 days before cell collection for subsequent analysis. An equivalent volume of DMSO was added to the control group. This experimental setup allowed for the analysis of the role of AKT signaling in macrophage polarization.

### 2.5 CCK-8 assay

RAW264.7 cells were uniformly seeded in 24-well plates and subjected to the intervention protocols described in this study. After treatment, 10 μL of CCK-8 reagent (HY-K0301, MCE, USA) was added to each well, followed by mixing and incubation for 2 hours. The absorbance at 450 nm (A_450_) was measured using a microplate reader. Cell viability was calculated as follows: Cell viability (%) = (A _treated_ - A _blank_)/(A _control_ - _A blank_) × 100%. Each experiment was performed in triplicate to assess the effects of different interventions on macrophage viability.

### 2.6 Western blot analysis

Total protein was extracted from gastrocnemius and RAW264.7 macrophages using pre-cooled RIPA buffer (Beyotime, China). Standard procedures included electrophoresis, membrane transfer, blocking, and incubation with the following primary antibodies at 4 °C overnight: iNOS (ab15323, Abcam, USA), Arg-1 (ab60176, Abcam, USA), AKT (9272, CST, USA), phospho-AKT(Ser^473^) (4060, CST, USA), β-tubulin (YM3030, Immunoway, China), β-actin (YM1207, Immunoway, China). Membranes were then incubated with species-appropriate secondary antibodies at 4 °C for 2 hours: goat anti-rabbit IgG (ZB5301, ZSGB-BIO, China) for iNOS, AKT, and phospho-AKT(Ser^473^); goat anti-mouse IgG (ZB5305, ZSGB-BIO, China) for Arg-1, β-tubulin, and β-actin. Then, the membranes were exposed using a ChemiDoc XRS imaging system (Bio-Rad, USA). Immobilon Western Chemiluminescent HRP substrate (Millipore, USA) was used to produce a signal to visualize the protein bands. The captured images were analyzed using Image-Pro plus 6.0 (Media Cybernetics, Inc., USA).

### 2.7 Immunofluorescence staining

Tissue sections were fixed, blocked, and incubated overnight at 4 °C with the following primary antibodies: GLUT4 (ab654, Abcam, USA), F4/80 (ab6640, abcam, USA), phospho-AKT(Ser^473^). Sections were then incubated with species-specific fluorescent secondary anti-bodies in the dark: goat anti-rabbit IgG (A0423, Beyotime, China) for GLUT4 and phospho-AKT(Ser^473^); goat anti-mouse IgG (A0521, Beyotime, China) for F4/80. Nuclei were counterstained with DAPI (D9542, Sigma, USA) for 10 minutes. Images were captured using a fluorescence microscope and analyzed with Image Pro Plus 6.0.

### 2.8 Hematoxylin and eosin Staining

Tissue sections were stained with hematoxylin for 5 minutes, rinsed, and sequentially dehydrated in 85% and 95% ethanol (5 minutes each). Subsequently, sections were stained with eosin for 5 minutes, followed by dehydration through absolute ethanol (three changes) and xylene (two changes), 5 minutes each. Finally, sections were mounted and examined under a microscope for image acquisition and analysis.

### 2.9 Enzyme-linked immunosorbent assay

Levels of MCP-1 (MJE00B, R&D SYSTEMS, USA), TNF-α (E-EL-M0049c, Elabscience, China), IL-1β (MLB00C, R&D SYSTEMS, USA), IL-6 (M600B, R&D SYSTEMS, USA), and IL-10 (ab108870, Abcam, USA) in skeletal muscle and serum were measured by ELISA. Samples (50 μg total protein per well) were processed according to the manufacturer’s instructions, and protein concentrations were calculated based on standard curves.

### 2.10 Flow cytometry

After the intervention above, RAW264.7 macrophages were dissociated with trypsin-EDTA into single cell suspension. They were washed and stained with anti-CD16/32 (553142, BD Biosciences, United States) for 20 min at room temperature to block Fc receptors before incubation with PE/Cy7-labelled anti-F4/80 (123114, BioLegend, USA), FITC-labelled anti-CD86 (105006, BioLegend, USA), and PE-labelled anti-CD206 (141706, BioLegend, USA) monoclonal antibodies in a dark room for 1 h. Subsequently, the cells were examined with flow cytometry (Beckman, United States). The expressions of cell markers were compared with an isotype control. The acquired flow cytometric images were analyzed with FlowJo program (version 10.0.7). The ratio of F4/80^+^ cells were used to quantify activated macrophage; the ratio of F4/80^+^CD86^+^CD206^-^ cells were used to quantify M1 macrophage; the ratio of F4/80^+^CD206^+^CD86^-^ cells were used to quantify M2 macrophage. The detailed gating strategy for macrophage identification and subset analysis is provided in **Supplementary Figure 1**.

### 2.11 Statistical analysis

Data are reported as mean ± SD. Statistical analyses were performed with GraphPad Prism software (version 8.0.1, San Diego, CA). The normality of distribution for each dataset was assessed using the Shapiro-Wilk test. Non-normal data or unequal variance were log transformed. The repeated-measures ANOVA was used to analyze the differences of blood glucose in mice and p-AKT/AKT in RAW264.7 cells. Comparisons between two independent groups were performed using the two-tailed Student’s t-test. The multi-group comparisons were performed with the two-way ANOVA test to assess main effects and interactions. In the case of a significant interaction, Bonferroni-corrected *post hoc* tests were applied to examine simple effects. If the interaction was not significant, the main effects were interpreted directly. For all analyses, a P-value of 0.05 was considered to be statistically significant.

## 3 Results

### 3.1 High-fat diet induces IR in mice

**Figure 2:**
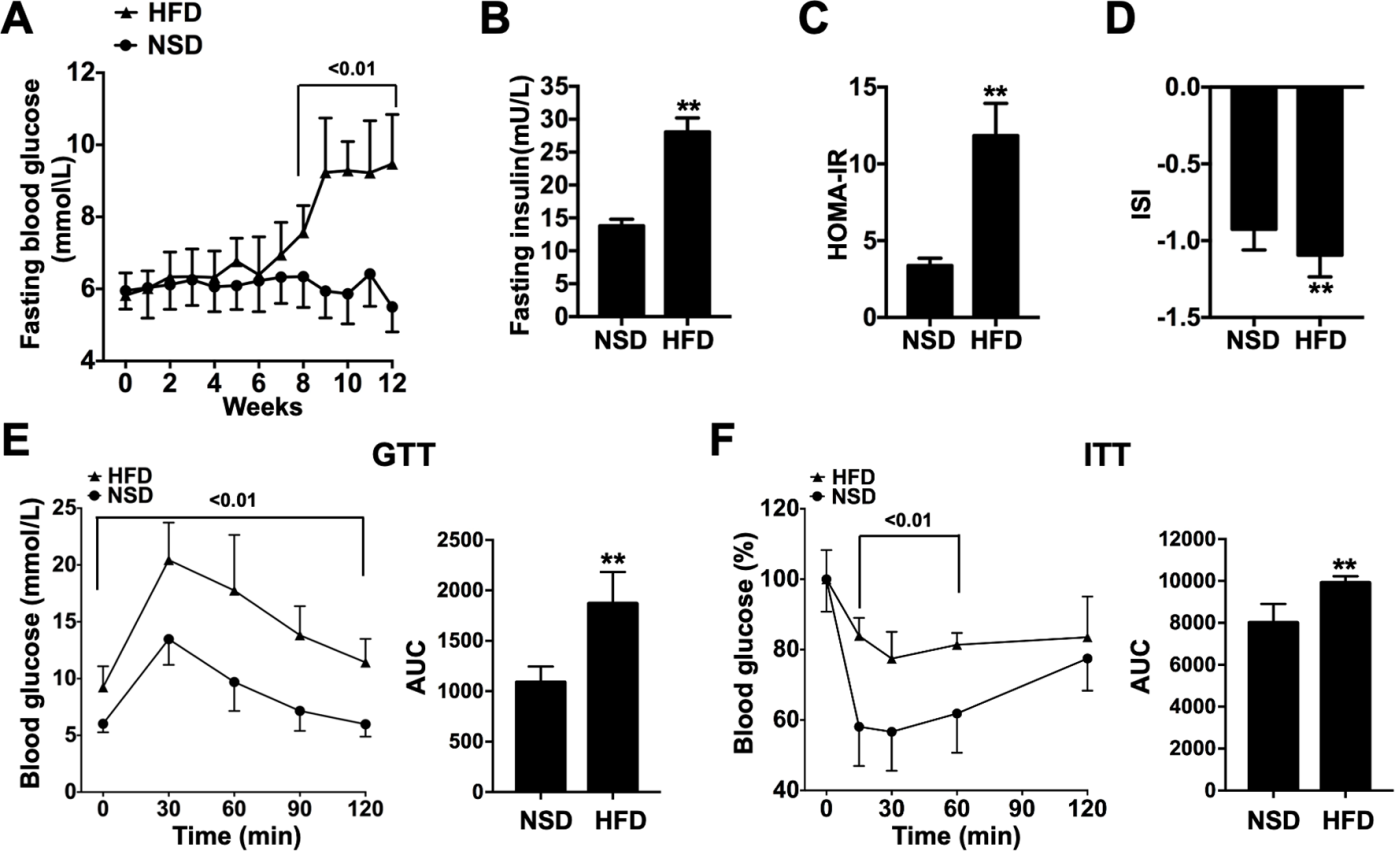
Metabolic alterations in insulin sensitivity following 12-week high-fat diet intervention in mice. **(A)** Fasting blood glucose levels, **(B)** Fasting insulin levels, **(C)** Homeostasis model assessment of insulin resistance, **(D)** Insulin sensitivity index, **(E)** Intraperitoneal glucose tolerance test, **(F)** Insulin tolerance test. Data were presented as mean ± SD. **p < 0.01 compared with NSD group.

Longitudinal monitoring of FBG levels during the modeling phase demonstrated that following 8 weeks of HFD intervention, the HFD group exhibited significant elevation in FBG compared to the NSD group at matched time points (p < 0.01, **Figure 2A**). Assessment of FINS levels and subsequent derivation of HOMA-IR and ISI revealed significantly increased FINS and HOMA-IR values (p < 0.01, **Figures 2B, C**), concomitant with a pronounced decrease in ISI (p < 0.01, **Figure 2D**) in HFD-fed mice. Moreover, GTT and ITT analyses confirmed impaired glucose homeostasis and insulin sensitivity in the HFD group, as reflected by right-shifted response curves and enlarged AUC in both assays (p < 0.01, **Figures 2E, F**). Taken together, these metabolic alterations substantiate the successful induction of IR in mice after 12 weeks of high-fat diet regimen.

### 3.2 Eccentric treadmill training ameliorates insulin sensitivity in skeletal muscle of insulin-resistant mice

**Figure 3:**
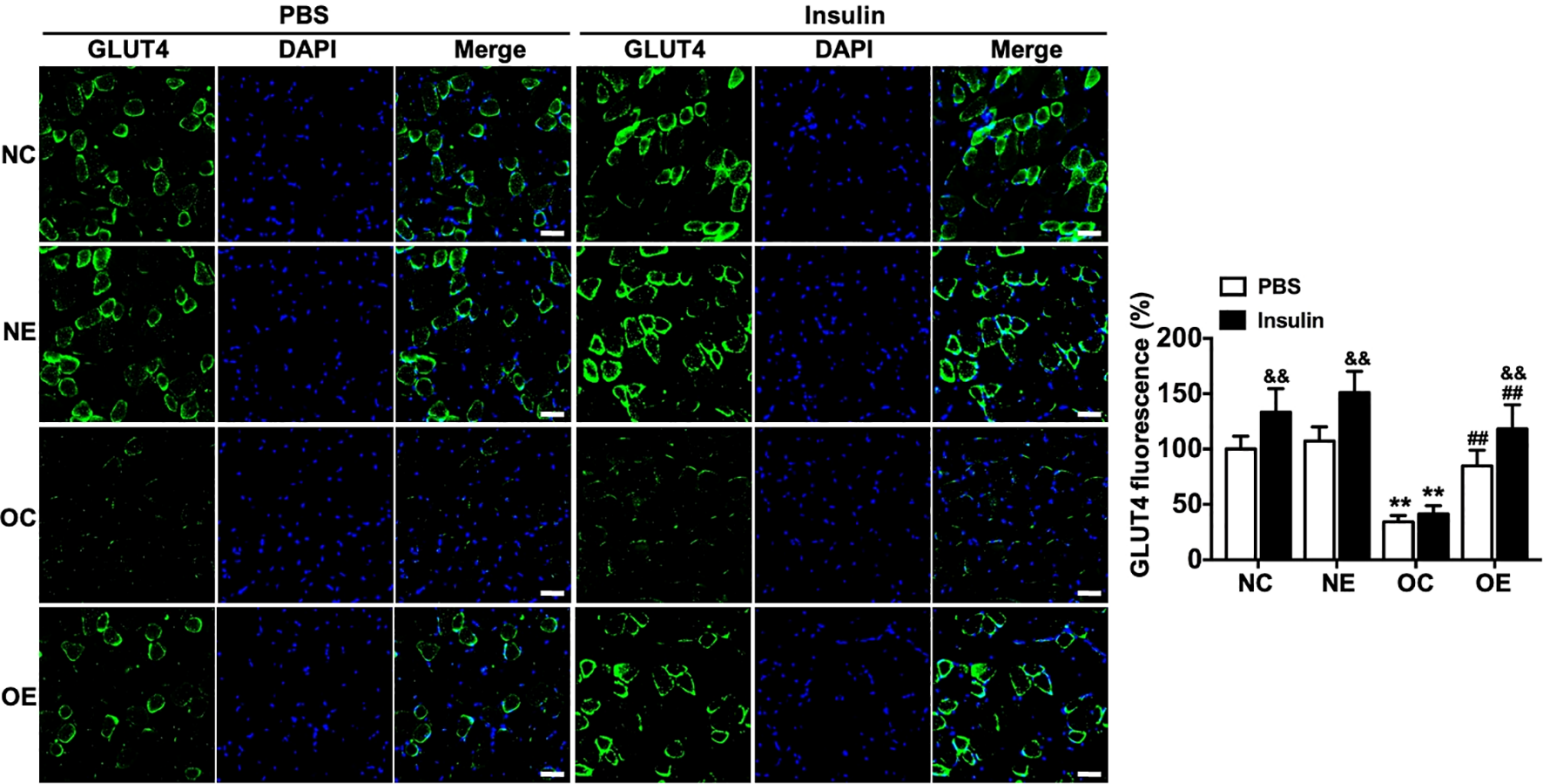
Alterations in GLUT4 distribution in skeletal muscle following eccentric treadmill training (scale bar = 50 μm, ×200 magnification). Data were presented as mean ± SD. **p < 0.01 compared with NC group; ##p < 0.01 compared with matched sedentary group; &&p < 0.01 compared with matched PBS group.

Immunofluorescence staining of GLUT4 revealed that insulin stimulation significantly increased GLUT4 fluorescence intensity in the NC, NE, and OE groups compared to their respective basal levels (p < 0.01). In contrast, the OC group showed no significant difference in GLUT4 fluorescence between insulin-stimulated and basal conditions (p > 0.05). The GLUT4 fluorescence intensity in the OC group was significantly lower than that in the NC group (p < 0.01), whereas 8-week eccentric treadmill training effectively elevated GLUT4 fluorescence in the OE group compared to the OC group (p < 0.01, **Figure 3**). These results demonstrate that high-fat diet intervention successfully induced IR in mouse skeletal muscle, and moderate-intensity eccentric treadmill exercise effectively ameliorated this condition.

### 3.3 Eccentric treadmill training attenuates skeletal muscle inflammation in insulin-resistant mice

**Figure 4:**
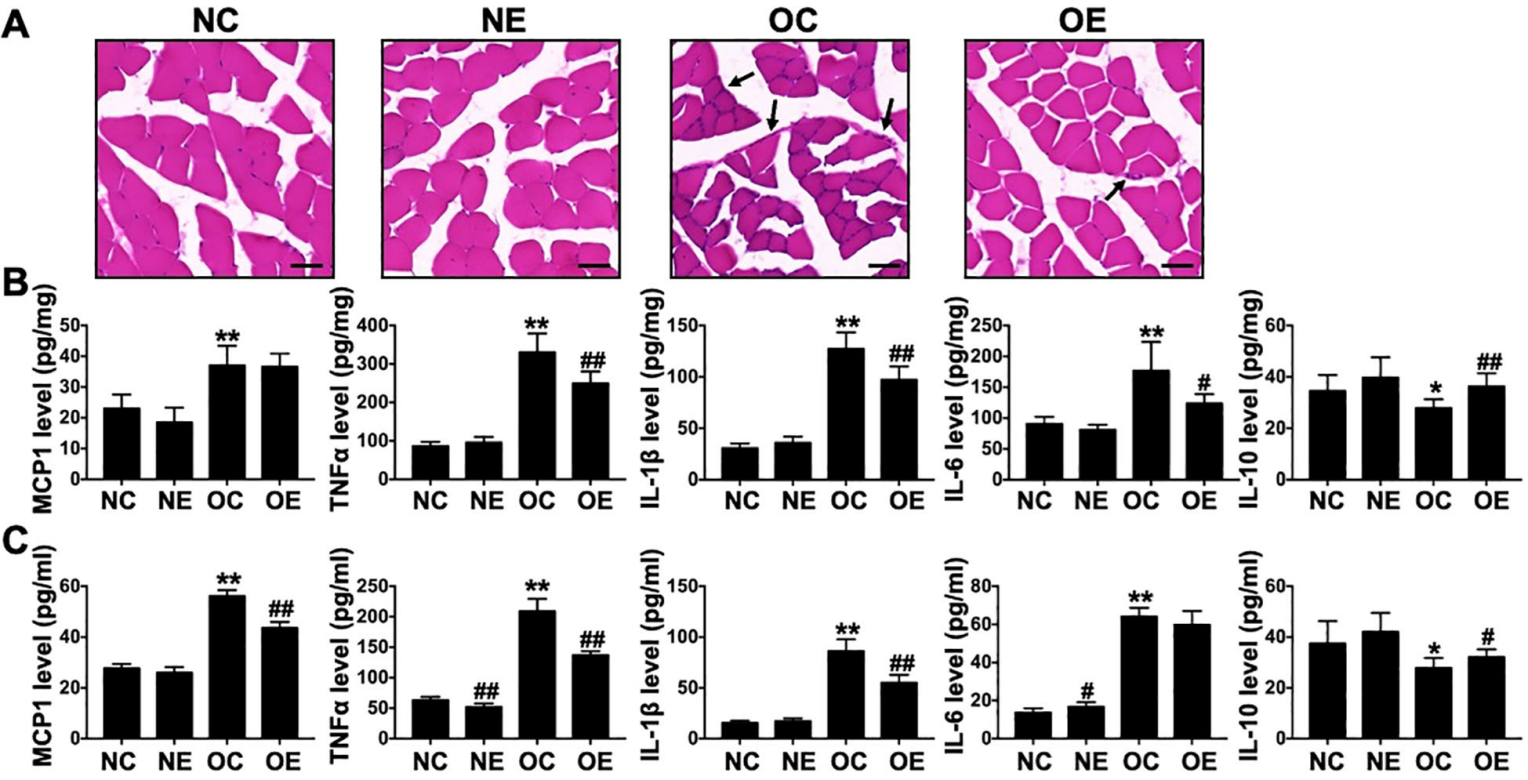
Systemic inflammatory responses in mice following eccentric treadmill training. **(A)** Representative H&E staining images of skeletal muscle tissue. Black arrows indicate infiltrated inflammatory cells (scale bar = 50 μm). **(B)** Alterations in inflammatory cytokine levels in skeletal muscle. **(C)** Changes in serum inflammatory cytokine profiles. Data were presented as mean ± SD. *p < 0.05, **p < 0.01 compared with NC group; #p < 0.05, ##p < 0.01 compared with matched sedentary group.

Histological analysis by H&E staining revealed substantial inflammatory cell infiltration in the skeletal muscle of OC group mice, whereas such infiltration was markedly reduced in the OE group (**Figure 4A**). Further quantification of inflammatory mediators demonstrated that the OC group exhibited significantly elevated of MCP-1, TNF-α, IL-1β, and IL-6, along with significantly reduced IL-10 levels, in both skeletal muscle (**Figure 4B**) and serum (**Figure 4C**) compared to the NC group (p < 0.05). Following eccentric training, the OE group showed significantly lower concentrations of TNF-α, IL-1β, and IL-6 (p < 0.05), and higher IL-10 levels (p < 0.01) in skeletal muscle compared to the OC group. In serum, the OE group exhibited significantly reduced MCP-1, TNF-α, and IL-1β (p < 0.01), and elevated IL-10 (p < 0.05). These findings indicate that high-fat diet feeding induces systemic chronic inflammation, and eccentric treadmill exercise effectively mitigates inflammatory responses in both skeletal muscle and circulation in IR mice.

### 3.4 Eccentric treadmill training modulates macrophage polarization in skeletal muscle of insulin-resistant mice

**Figure 5:**
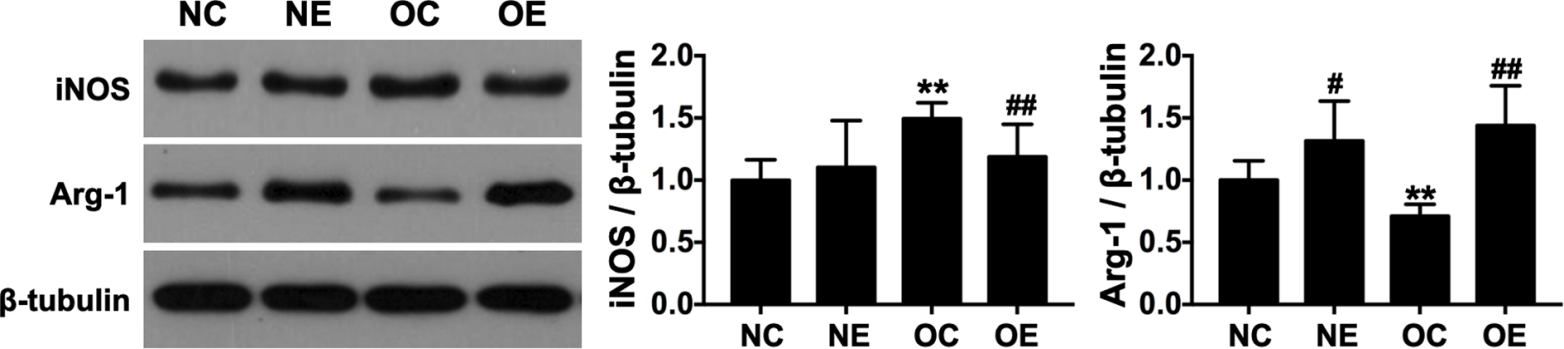
Macrophage polarization levels in skeletal muscle following eccentric treadmill training. Data were presented as mean ± SD. **p < 0.01 compared with NC group; #p < 0.05, ##p < 0.01 compared with matched sedentary group.

Analysis of macrophage polarization markers in skeletal muscle revealed significantly elevated iNOS (an M1 marker) and reduced Arg-1 (an M2 marker) expression in the OC group compared to the NC group (p < 0.01). Eccentric treadmill training effectively reversed this polarization pattern, with the OE group exhibiting significantly lower iNOS and higher Arg-1 expression relative to the OC group (p < 0.01, **Figure 5**). These results suggest that eccentric exercise intervention attenuates high-fat diet-induced M1 macrophage polarization while promoting an M2-polarized phenotype in skeletal muscle.

### 3.5 Potential mechanisms by which eccentric treadmill training modulates skeletal muscle macrophage polarization

**Figure 6:**
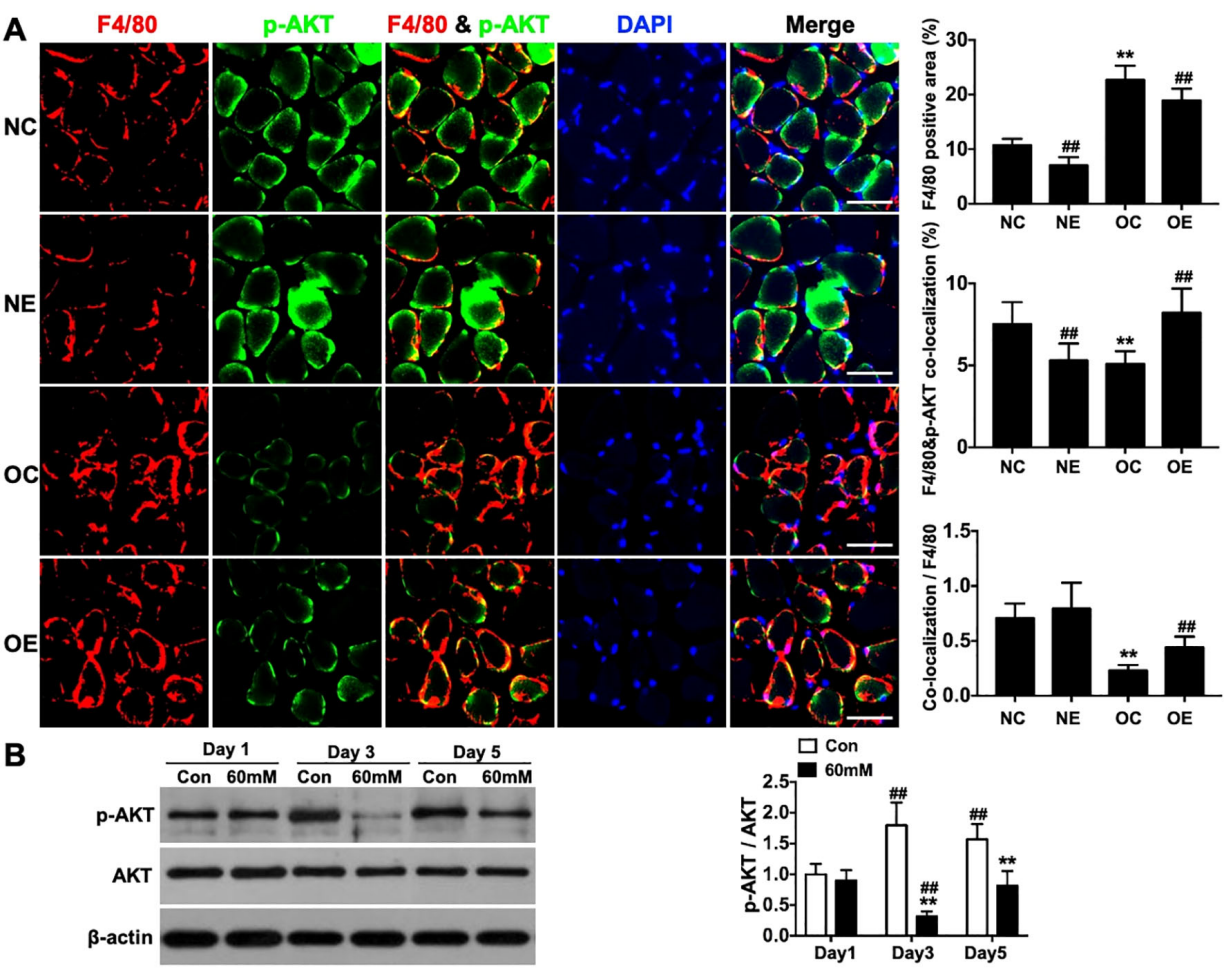
phospho-AKT(Ser^473^) levels in macrophages following *in vivo* eccentric treadmill training or *in vitro* high glucose intervention. **(A)** Co-localization of F4/80 and phospho-AKT(Ser^473^) in skeletal muscle (scale bar = 50 μm, ×400 magnification). **(B)** phospho-AKT(Ser^473^) levels in macrophages under *in vitro* conditions. Data were presented as mean ± SD. **p < 0.01 compared with NC group; ##p < 0.01 compared with matched sedentary group **(A)**. **p < 0.01 compared with Con group at the same time point; ##p < 0.01 compared with Day 1 within the same group **(B)**.

Immunofluorescence analysis revealed significantly decreased F4/80&phospho-AKT(Ser^473^) co-localization area and co-localization-to-F4/80 ratio in the OC group compared to the NC group (p < 0.01). These parameters were significantly elevated in the OE group relative to the OC group (p < 0.01, **Figure 6A**). *In vitro* experiments showed that high glucose treatment for 3 and 5 days significantly reduced the phospho-AKT(Ser^473^)/AKT ratio in the 60mM group compared to the Con group (p < 0.01, **Figure 6B**).

**Figure 7:**
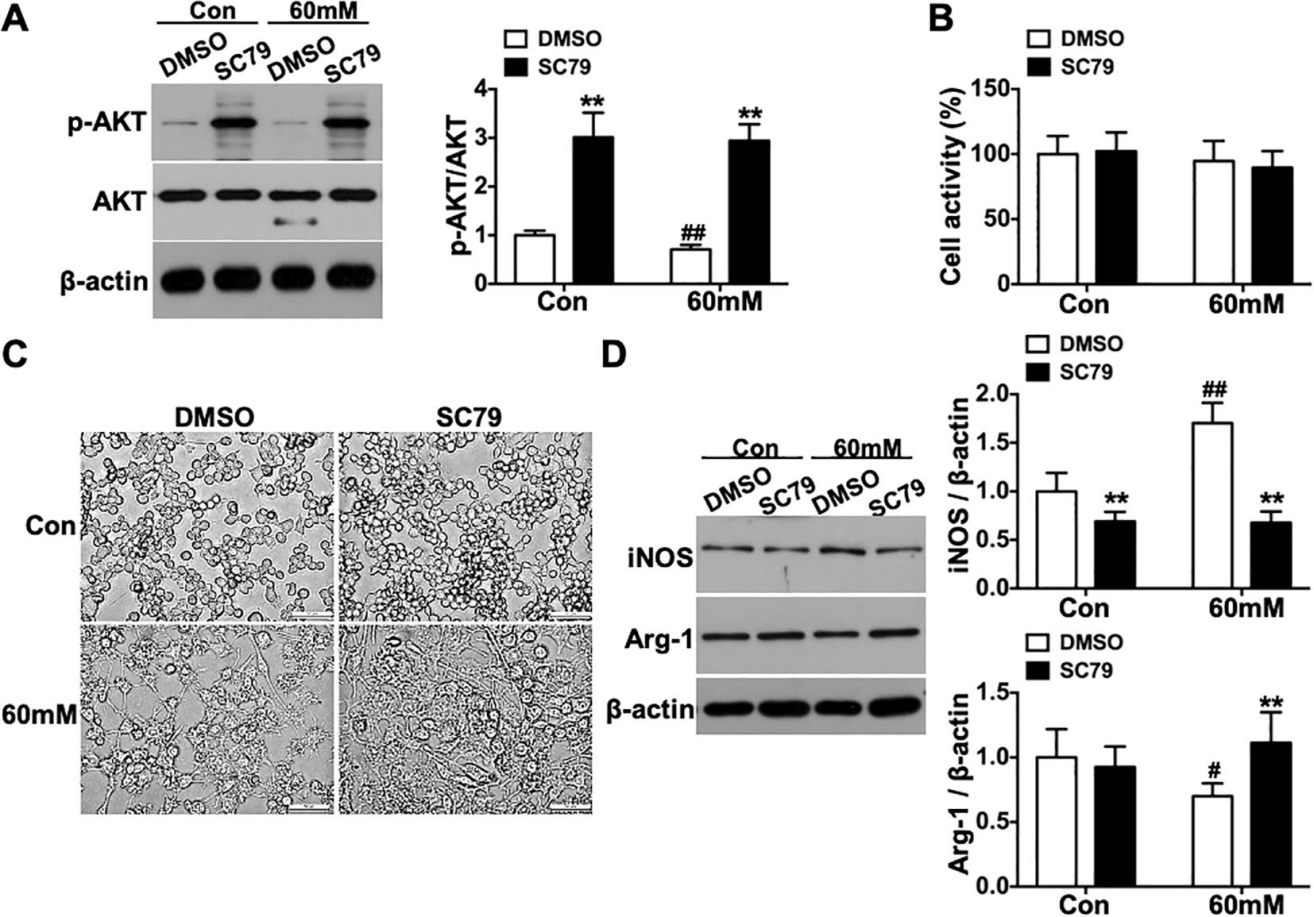
Polarization status of RAW264.7 macrophages under high glucose conditions following AKT activation *in vitro*. **(A)** phospho-AKT(Ser^473^) levels. **(B)** Changes in cell viability. **(C)** Morphological alterations of cells (scale bar = 50 μm, ×400 magnification). **(D)** Protein expression levels of iNOS and Arg-1. Data were presented as mean ± SD. **p < 0.01 compared with matched DMSO group; #p < 0.05, ##p < 0.01 compared with matched Con group.

SC79 (AKT agonist) treatment significantly increased phospho-AKT(Ser^473^)/AKT levels in both the 60mM and Con groups compared to their corresponding DMSO controls (p < 0.01, **Figure 7A**), with no significant difference observed between the SC79-treated 60mM and Con groups (p > 0.05). SC79 treatment under high glucose conditions did not significantly affect macrophage viability (p > 0.05, **Figure 7B**). Morphological analysis showed that high glucose conditions induced elongated and irregular macrophage shapes, while SC79 treatment promoted a transition to rounded morphology (**Figure 7C**). Western blot analysis demonstrated that SC79 significantly suppressed high glucose-induced iNOS expression (p < 0.01) and enhanced Arg-1 expression (p < 0.01, **Figure 7D**).

**Figure 8:**
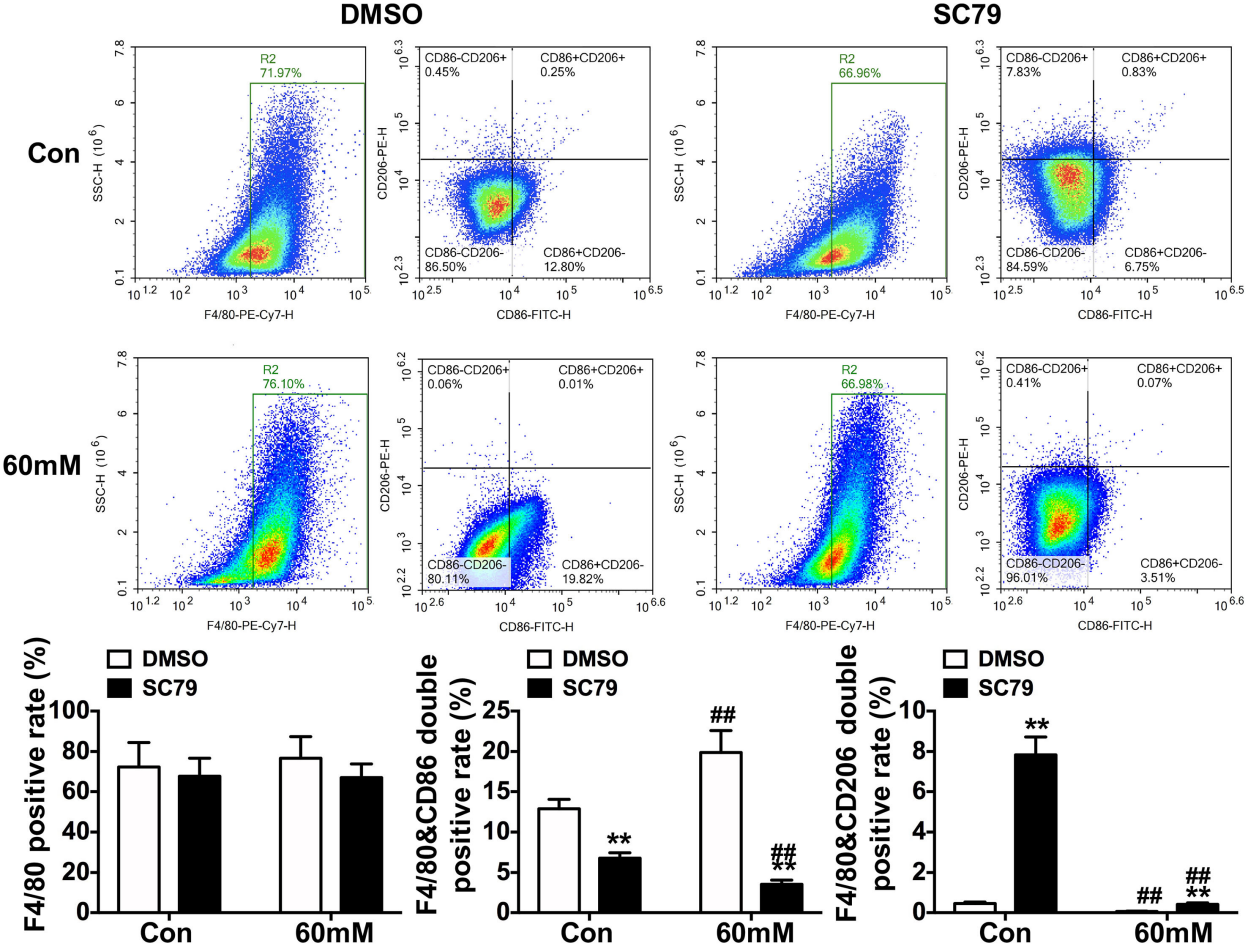
Alterations in surface polarization markers of RAW264.7 macrophages under high glucose conditions following AKT activation *in vitro*. Data were presented as mean ± SD. **p < 0.01 compared with matched DMSO group; ##p < 0.01 compared with matched Con group.

Flow cytometry further confirmed that SC79 significantly reduced the F4/80&CD86 double-positive rate (M1 marker) and increased the F4/80&CD206 double-positive rate (M2 marker) in both groups (p < 0.01, **Figure 8**).

**Figure 9:**
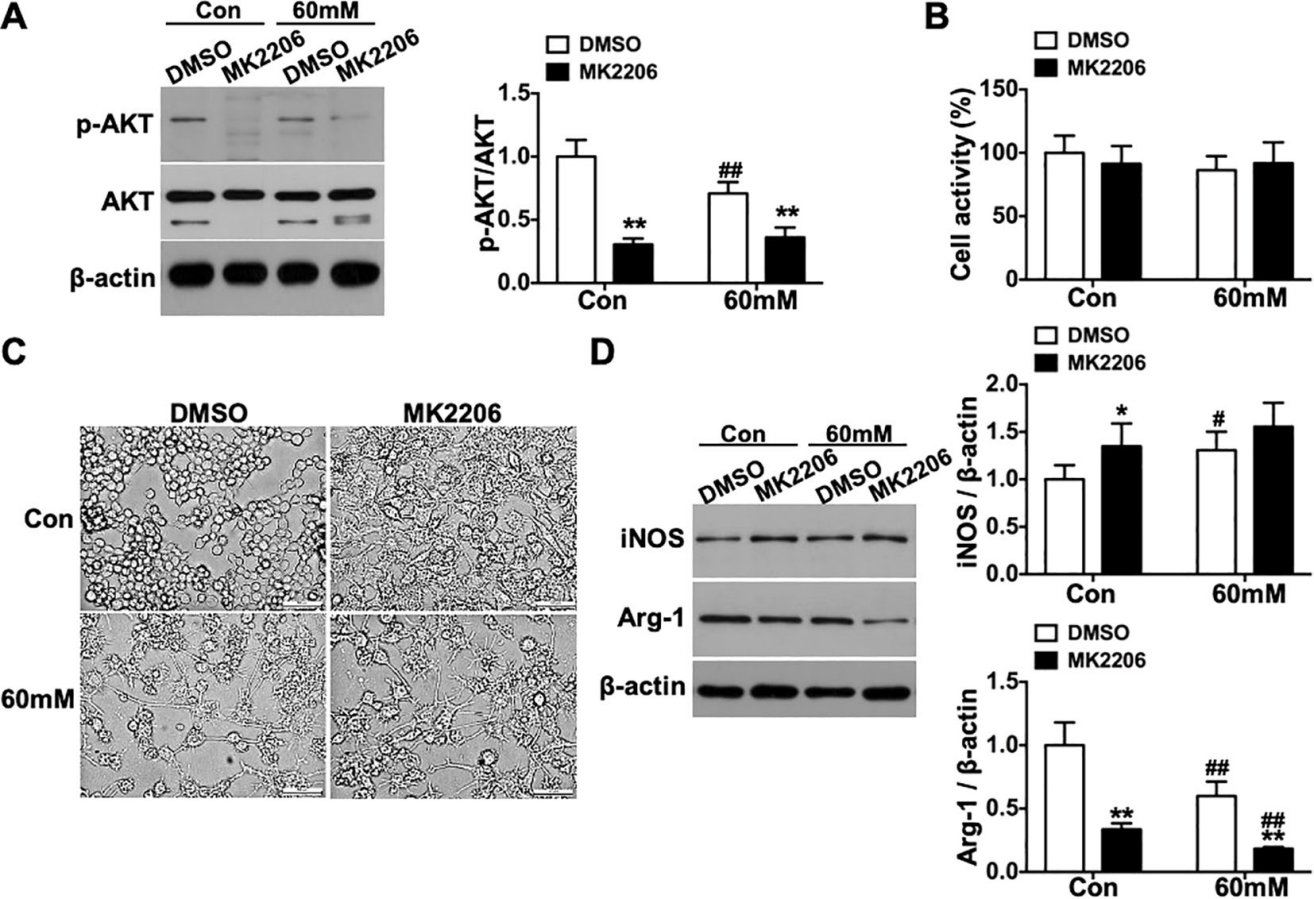
Polarization status of RAW264.7 macrophages under high glucose conditions following AKT inhibition *in vitro*. **(A)** phospho-AKT(Ser^473^) levels. **(B)** Changes in cell viability. **(C)** Morphological alterations of cells (scale bar = 50 μm, ×400 magnification). **(D)** Protein expression levels of iNOS and Arg-1. Data were presented as mean ± SD. *p < 0.05, **p < 0.01 compared with matched DMSO group; #p < 0.05, ##p < 0.01 compared with matched Con group.

Conversely, MK2206 (AKT inhibitor) treatment significantly decreased phospho-AKT(Ser^473^)/AKT levels in both the 60mM and Con groups (p < 0.01, **Figure 9A**) without affecting cell viability (p > 0.05, **Figure 9B**). MK2206 treatment exacerbated the elongated and irregular morphological changes (**Figure 9C**), increased iNOS expression (p < 0.05 in Con group), and suppressed Arg-1 expression in both groups (p < 0.01, **Figure 9D**).

**Figure 10:**
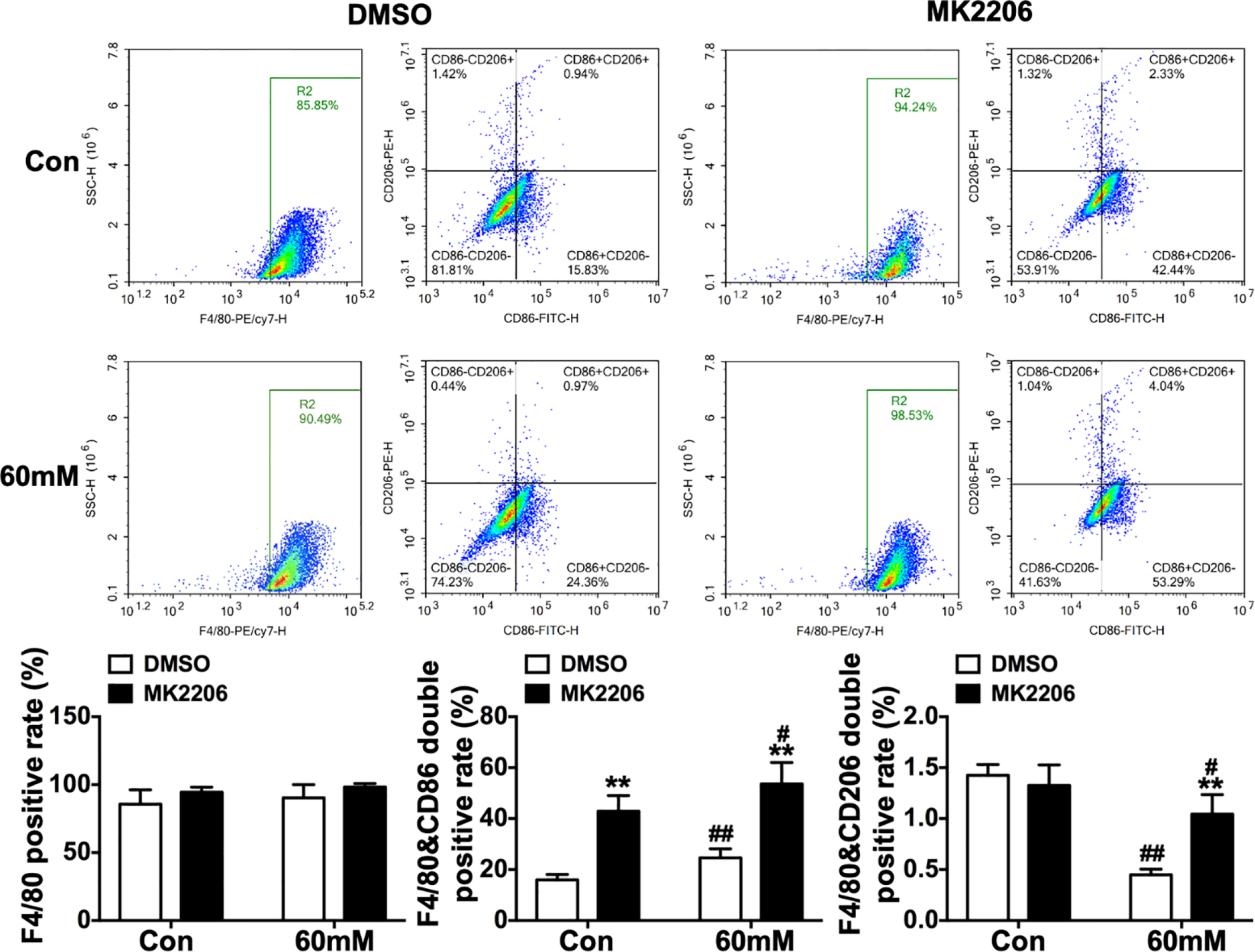
Alterations in surface polarization markers of RAW264.7 macrophages under high glucose conditions following AKT inhibition *in vitro*. Data were presented as mean ± SD. **p < 0.01 compared with matched DMSO group; #p < 0.05, ##p < 0.01 compared with matched Con group.

Flow cytometry analysis showed that MK2206 significantly increased the F4/80&CD86 double-positive rate in both groups (p < 0.01, **Figure 10**). Collectively, these results indicate that phospho-AKT(Ser^473^) activation alleviates high glucose-induced M1 macrophage polarization while promoting M2 polarization, whereas AKT inhibition exacerbates M1 polarization and suppresses M2 polarization.

## 4 Discussion

IR serves as a common pathological basis for various metabolic diseases (30, 31). Due to limited clinical intervention efficacy, research on exercise interventions and their underlying mechanisms has become a frontier topic of mutual interest in both sports’ science and healthcare, holding significant importance for early prevention and management of metabolic diseases (32–34). In recent years, repeated eccentric exercise has garnered increasing interest as a potentially superior intervention for ameliorating IR, owing to its characteristic profile of low metabolic demand coupled with high mechanical output (7–10). However, the underlying mechanisms responsible for these effects remain incompletely understood. Therefore, the present study employed a moderate-intensity treadmill running protocol at a -5° slope in a high-fat diet-induced IR mouse model, with a specific focus on skeletal muscle macrophage polarization and AKT signaling pathway activation. Through integrated *in vivo* and *in vitro* experiments, we investigated the therapeutic effects and potential mechanisms of eccentric treadmill training on skeletal muscle IR. Our findings provide novel insights into the mechanisms through which repeated eccentric exercise ameliorates IR, offering new perspectives and methodologies for optimizing exercise interventions in metabolic diseases and establishing a theoretical foundation for exercise prescriptions targeting IR and type 2 diabetes.

Numerous studies have demonstrated that, under equivalent cardiovascular load, skeletal muscle eccentric contractions generate 4–5 times the power output of concentric contractions, enabling greater work volume and imposing stronger stimuli on skeletal muscle (7–10). Consequently, moderate-intensity eccentric exercise may represent a more effective approach for ameliorating skeletal muscle IR compared to concentric exercise. For instance, Chen et al. (11) demonstrated that 12 weeks of downhill walking and eccentric knee extension resistance exercise more effectively improved skeletal muscle strength and systemic insulin sensitivity in obese elderly individuals compared to concentric exercise, without inducing DOMS, highlighting the need for greater research focus on eccentric exercise in sports medicine. Similarly, Julian et al. (8) reported that 12 weeks of eccentric cycling more significantly enhanced skeletal muscle mass and function, thereby improving IR in obese adolescents, suggesting eccentric exercise as an optimal modality for obese populations. Philippe et al. (12) proposed that 3 weeks of downhill exercise (predominantly eccentric) yielded superior improvements in glycolipid metabolism and chronic inflammation compared to uphill exercise in prediabetic individuals. In our study, 12 weeks of high-fat diet intervention significantly increased FBG and FINS levels, elevated HOMA-IR, reduced ISI, and increased AUC values in GTT and ITT, confirming successful induction of IR in mice. Focusing on skeletal muscle, we observed loss of plasma membrane translocation responsiveness to insulin stimulation in the key glucose transporter GLUT4 following high-fat diet intervention, indicating skeletal muscle IR. Importantly, 8 weeks of moderate-intensity eccentric treadmill training effectively restored insulin-stimulated GLUT4 translocation in skeletal muscle of IR mice, demonstrating that such training significantly enhances skeletal muscle insulin sensitivity.

Evidence suggests that energy surplus-induced chronic inflammation is closely linked to IR, with the two conditions coexisting and mutually reinforcing each other, collectively contributing to metabolic disease pathogenesis (35, 36). Under metabolic dysregulation, increased immune cell infiltration into tissues and subsequent pro-inflammatory cytokine release exacerbate inflammatory responses and metabolic disturbances, accelerating IR progression and ultimately leading to diabetes and other metabolic disease (37, 38). Macrophages, as crucial immune cells and central mediators of chronic inflammation, can polarize into functionally distinct pro-inflammatory M1 or anti-inflammatory M2 phenotypes in response to microenvironmental cues, thereby playing pivotal roles in regulating inflammation and metabolism (16, 17). Current research confirms that uncontrolled M1 macrophage polarization in adipose tissue mediates obesity-associated IR: under physiological conditions, early inflammation is dominated by M1 polarization, promoting inflammatory responses, followed by increased M2 polarization and M1-to-M2 transition, which suppresses inflammation and facilitates tissue repair. In obesity, adipose tissue exhibits enhanced macrophage infiltration with sustained M1 polarization and impaired M2 polarization, leading to M1/M2 imbalance, chronic inflammation, and IR (17). Our previous studies also demonstrated increased M1 and decreased M2 polarization in adipose tissue of IR mice (18), while *in vitro* high glucose treatment induced M1 polarization in RAW264.7 macrophages (19). In skeletal muscle, substantial evidence indicates that metabolic stress triggers macrophage infiltration into muscle tissue, participating in inflammatory-metabolic crosstalk: uncontrolled M1 polarization induces chronic inflammation and metabolic dyshomeostasis, whereas M2 polarization exerts anti-inflammatory effects and promotes metabolic balance restoration (20, 21). For example, Brestoff et al. (20) proposed that macrophages in healthy individuals promote tissue glucose utilization and maintain lipid homeostasis, whereas obesity-induced M1 polarization triggers chronic inflammation and metabolic dysregulation. De Santa et al. (21) suggested that modulating the tissue microenvironment to suppress skeletal muscle M1 polarization may represent an effective therapeutic strategy for metabolic diseases. Our previous *in vitro* research demonstrated that high glucose-induced macrophage M1 polarization triggers IR in skeletal muscle myoblasts (19). Consistent with these findings, our current *in vivo* study revealed that high-fat diet intervention increased inflammatory cell infiltration, enhanced M1 polarization, and reduced M2 polarization in mouse skeletal muscle, creating a chronic inflammatory state that contributes to skeletal muscle IR.

Although the role of macrophage polarization in moderate-intensity eccentric exercise-mediated improvement of skeletal muscle IR remains incompletely understood, substantial evidence confirms that repeated eccentric exercise increases skeletal muscle macrophage infiltration while reducing inflammatory responses (39–41). According to Scala et al. (39), the primary mechanism underlying the post-exercise increase in muscle macrophages involves the recruitment of circulating monocytes into muscle tissue in response to exercise-induced stress. These monocytes subsequently differentiate into macrophages, initially adopting a pro-inflammatory (M1) phenotype. Zuo et al. (40) observed increased M1 polarization immediately after eccentric exercise, followed by M2 polarization elevation and reduced muscle inflammation at 12 hours post-exercise, suggesting dynamic macrophage phenotype transitions regulate inflammatory progression and metabolism in skeletal muscle. Hyldahl et al. (22) and Peake et al. (23) proposed that repeated eccentric exercise accelerates the transition from M1 to M2 polarization, alleviating inflammation and promoting repair. Deyhle et al. (9) found that repeated eccentric exercise increased macrophage infiltration while significantly reducing DOMS, suggesting that modulation of macrophage polarization may underlie inflammation improvement and tissue repair. Huang et al. (41) further demonstrated that inhibiting macrophage infiltration disrupts post-eccentric exercise inflammatory progression and induces muscle atrophy, validating the essential role of macrophages in exercise-mediated inflammation regulation. In our study, 8 weeks of moderate-intensity eccentric treadmill training significantly reduced M1 polarization and enhanced M2 polarization in skeletal muscle of IR mice. Concurrently, we observed decreased pro-inflammatory cytokine levels and increased anti-inflammatory cytokine levels in both skeletal muscle and serum, indicating effective amelioration of chronic inflammation. These findings suggest that moderate-intensity eccentric treadmill training may improve IR by suppressing M1 polarization and promoting M2 polarization, thereby reducing skeletal muscle inflammation.

The PI3K/AKT signaling pathway represents a highly evolved and conserved pathway involved in various cellular processes including proliferation, apoptosis, and differentiation (42, 43). Accumulating evidence confirms that AKT signaling serves as a key regulator of macrophage polarization and function (44, 45). For example, Zhao et al. (44) demonstrated significantly enhanced AKT signaling in M2 macrophages compared to M1 phenotypes, and AKT inhibition substantially impaired M2 polarization. Yu et al. (45) reported that insulin promotes M1-to-M2 transition in diabetic rats through AKT phosphorylation activation. In our study, *in vivo* experiments revealed decreased phospho-AKT(Ser^473^) in skeletal muscle macrophages of IR mice, associated with promoted M1 and suppressed M2 polarization. Similarly, studies *in vitro* showed reduced phospho-AKT(Ser^473^) in macrophages under high glucose conditions. These results collectively suggest that AKT signaling plays a crucial role in high glucose-induced M1 polarization in IR.

Although the role of AKT signaling in eccentric exercise-mediated regulation of macrophage polarization remains unclear, numerous studies demonstrate that exercise interventions effectively upregulate AKT phosphorylation (46–48). For instance, Zhang et al. (46) demonstrated that exercise promotes hypertrophy of type II muscle fibers by activating the IGF-1/AKT/mTORC1 signaling pathway, which in turn mitigates both sarcopenia and adiposity. Zhao et al. (47) found that treadmill exercise promotes axon regeneration and improves motor function via the activation of the IGF-1R/AKT/mTOR signaling pathways. Xiaoyu et al. (48) demonstrated that exercise rehabilitation activates the BDNF/TrkB/PI3K/AKT signaling pathway, thereby effectively reducing neuronal apoptosis and inflammatory responses following intracerebral hemorrhage in mice. In our research, 8 weeks of moderate-intensity eccentric treadmill training significantly enhanced phospho-AKT(Ser^473^) in skeletal muscle macrophages of IR mice, promoting M2 polarization while suppressing M1 polarization, ultimately reducing systemic inflammation and improving skeletal muscle IR. To further elucidate the critical regulatory role of AKT signaling in macrophage polarization under high glucose conditions, we conducted AKT interventions in high glucose-treated macrophages. Results showed that while high glucose environment promoted M1 and suppressed M2 polarization, AKT activation via agonist treatment abolished these effects, increasing M2 polarization and reducing M1 polarization. Conversely, AKT inhibition further enhanced M1 polarization, confirming the pivotal role of AKT signaling in macrophage polarization regulation. These findings collectively demonstrate that AKT signaling mediates macrophage polarization, and moderate-intensity eccentric treadmill training likely activates AKT signaling in macrophages to suppress M1 while promoting M2 polarization, thereby reducing inflammation and improving skeletal muscle insulin sensitivity in IR mice. Although some studies indicated that PI3K/AKT signaling may inhibit the effects of anti-inflammatory cytokines and has a detrimental function in the process of macrophage activation under acute inflammatory stimuli such as acute pneumonia (49), sustained AKT activation-as observed following chronic eccentric training in our model-can promote alternative M2 polarization under physiological conditions (50–53). This context−dependent function highlights the plasticity of PI3K/AKT signaling and its potential to exert beneficial effects in metabolic disease when modulated by physiological interventions such as exercise.

## 5 Conclusions

In summary, this study revealed that moderate-intensity eccentric treadmill training effectively ameliorates skeletal muscle IR induced by high-fat diet. This improvement is mediated through a coordinated mechanism wherein eccentric exercise promotes phospho-AKT(Ser^473^) in skeletal muscle macrophages, leading to a phenotypic shift from pro-inflammatory M1 toward anti-inflammatory M2 polarization. The resultant alteration in macrophage polarization status subsequently attenuates local and systemic inflammation, ultimately restoring insulin sensitivity in skeletal muscle.

Our findings thus establish a mechanistic link between eccentric exercise and metabolic improvement, positioning AKT-mediated macrophage polarization as a key physiological pathway through which structured eccentric training counteracts diet-induced IR. It should be noted that the current study only tested expression of specific surface markers and cytokine profiles as indicators of macrophage polarization, without direct functional validation relevant to muscle physiology such as efferocytosis or clearance of debris. Therefore, further investigations are required to directly confirm macrophage polarization in this context. Furthermore, this work is limited to *in vitro* cellular-level analysis of AKT signaling in macrophage polarization. The critical role of macrophages and their AKT signaling in eccentric exercise-mediated improvement of skeletal muscle IR requires further *in vivo* validation. This represents an important direction for future research.

## Data Availability

The original contributions presented in the study are included in the article/**Supplementary Material**. Further inquiries can be directed to the corresponding author.
