# Supplementary material for: Eccentric treadmill training and skeletal muscle immunometabolic responses in HFD-induced insulin resistance
Source: Front Immunol. 2026 Feb 25;17:1757925. doi: 10.3389/fimmu.2026.1757925 (PMC12975572; doi:10.3389/fimmu.2026.1757925)
Supplement: Supplementary file 1 [file Supplementaryfile1.docx]

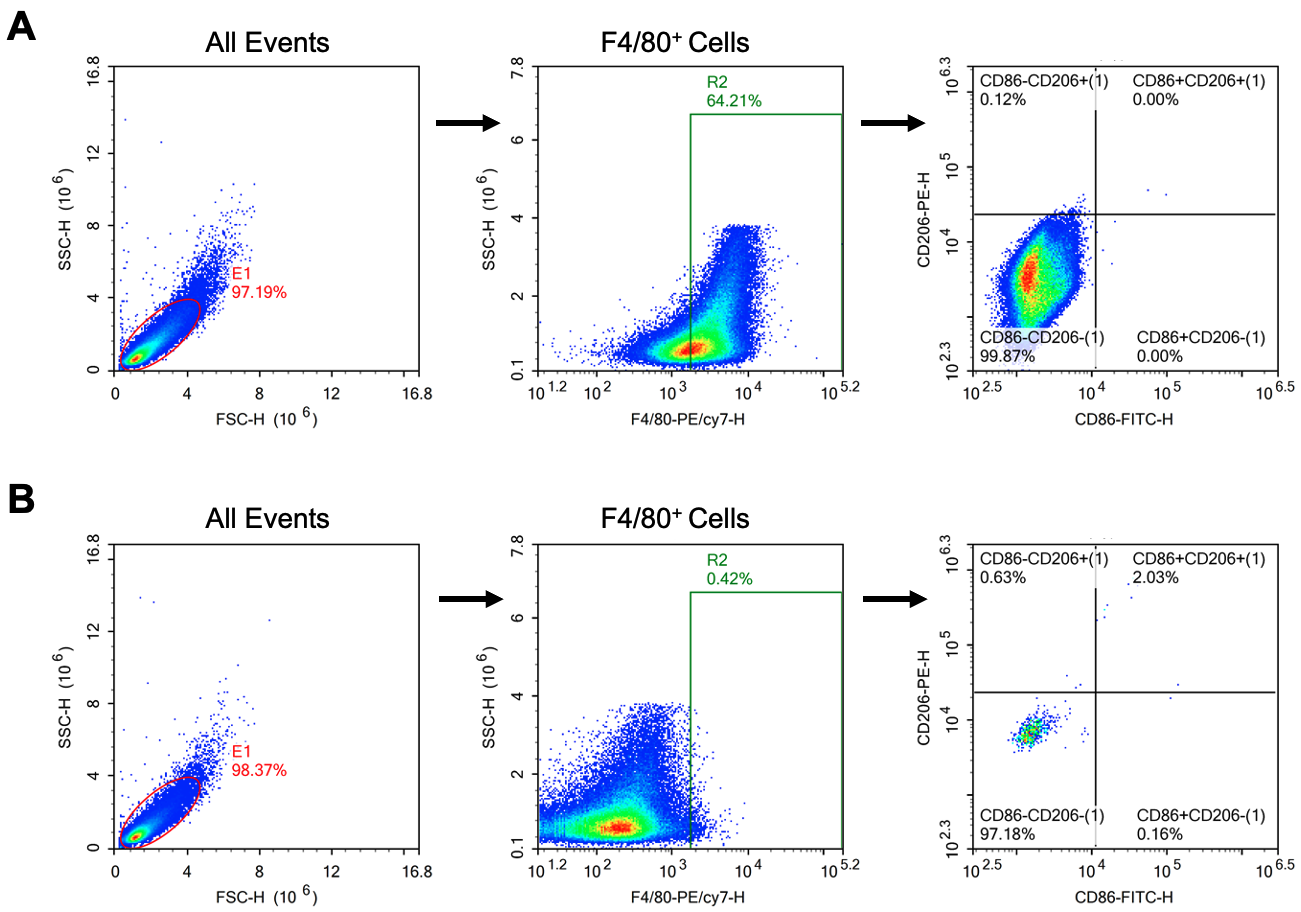


**Supplemental Figure S1:** Flow cytometry gating scheme to isolate F4/80^+^ macrophage. Gating strategy from RAW264.7 cell suspensions using pseudocolor defaults both A) F4/80 single-stain positive control and B) negative control. Macrophage labeling (F4/80^+^), M1 macrophage labeling (F4/80^+^CD86^+^ CD206^-^), and M2 macrophage labeling (F4/80^+^CD206^+^ CD86^-^) in the RAW264.7 cells.
